# AMG193: Discovery and Structural basis for MTA cooperative inhibition of PRMT5*(Work done at Amgen with PRMT5 Team)*

**DOI:** 10.1063/4.0001022

**Published:** 2025-10-27

**Authors:** Susmith Mukund

**Affiliations:** Independent Consultant, Foster City, CA, USA

## Abstract

The methyl thioadenosine phosphorylase (MTAP) gene which is proximal to the CDK2N2A tumor suppressor gene on chromosome locus p23q is frequently deleted in ∼15% of cancer cells. This results in the accumulation of methylthioadenosine (MTA), which competes with *S*-adenosyl methionine (SAM), the methyl donor for the essential enzyme, protein arginine methyltransferase 5 (PRMT5). PRMT5 is thereby put in a hypomorphic state in these MTAP-deleted cancer cells, presenting an opportunity for its further inhibition with MTA-cooperative inhibitors. DNA- encoded library screening produced hits that cooperatively bound PRMT5:MEP50 and MTA. Optimization of these compounds and structural enablement through both crystallography and cryo-electron microscopy led to the discovery of AMG 193.

Crystal structure shows AMG 193 occupying the peptide binding site of the PRMT5 catalytic domain, where the R3 of the peptide substrate binds during the catalytic cycle, and in the vicinity of the co-inhibitor MTA (see figure, PDB id: 9C10). The tricyclic dihydrofuro- naphthyridine warhead mimics the R^3^ of the substrate with similar interactions, its amino-heterocyclic moiety forming salt bridge with E^444^ acid and a H-bond with the backbone carbonyl of E^435^, the furan oxygen in a H-bond with K^333^. The amino-heterocycle is in a displaced π:π stacking between W^579^ and F^327^, as well as van der Waals contact with residue Glu^435^, and MTA, a key feature of the MTA cooperative nature of the inhibitor. AMG 193 is further stabilized by a H-bond between its amide carbonyl, which is perpendicular to the tricyclic core, and the backbone amide of F^580^. The morpholine substituent adopts an axial conformation relative to tricyclic warhead. The terminal trifluoro-phenyl is packed in the very hydrophobic distal end of the substrate pocket, F^580^ in a π:π stack and Y304 in an edge-to-face stack with the phenyl ring of the ligand. The structure further explains why AMG 193 is MTA-cooperative and not synergistic with SAM.

